# Structural progression of Alzheimer’s disease over decades: the MRI staging scheme

**DOI:** 10.1093/braincomms/fcac109

**Published:** 2022-04-28

**Authors:** Vincent Planche, José V. Manjon, Boris Mansencal, Enrique Lanuza, Thomas Tourdias, Gwenaëlle Catheline, Pierrick Coupé

**Affiliations:** Univ. Bordeaux, CNRS, UMR 5293, Institut des Maladies Neurodégénératives, F-33000 Bordeaux, France; Centre Mémoire Ressources Recherches, Pôle de Neurosciences Cliniques, CHU de Bordeaux, F-33000 Bordeaux, France; Instituto de Aplicaciones de las Tecnologías de la Información y de las Comunicaciones Avanzadas (ITACA), Universitat Politècnica de València, Camino de Vera s/n, 46022 Valencia, Spain; Univ. Bordeaux, CNRS, UMR5800, Bordeaux INP, LABRI, F-33400 Talence, France; Department of Cell Biology, University of Valencia, Burjassot 46100, Valencia, Spain; Inserm U1215 - Neurocentre Magendie, Bordeaux F-33000, France; Service de Neuroimagerie diagnostique et thérapeutique, CHU de Bordeaux, F-33000 Bordeaux, France; Institut de Neurosciences Cognitives et Intégratives d’Aquitaine, CNRS, UMR 5287, University of Bordeaux, F-33000 Bordeaux, France; Univ. Bordeaux, CNRS, UMR5800, Bordeaux INP, LABRI, F-33400 Talence, France

**Keywords:** Alzheimer, MRI, atrophy, staging, lifespan

## Abstract

The chronological progression of brain atrophy over decades, from pre-symptomatic to dementia stages, has never been formally depicted in Alzheimer’s disease. This is mainly due to the lack of cohorts with long enough MRI follow-ups in cognitively unimpaired young participants at baseline. To describe a spatiotemporal atrophy staging of Alzheimer’s disease at the whole-brain level, we built extrapolated lifetime volumetric models of healthy and Alzheimer’s disease brain structures by combining multiple large-scale databases (*n* = 3512 quality controlled MRI from 9 cohorts of subjects covering the entire lifespan, including 415 MRI from ADNI1, ADNI2 and AIBL for Alzheimer’s disease patients). Then, we validated dynamic models based on cross-sectional data using external longitudinal data. Finally, we assessed the sequential divergence between normal aging and Alzheimer’s disease volumetric trajectories and described the following staging of brain atrophy progression in Alzheimer’s disease: (i) hippocampus and amygdala; (ii) middle temporal gyrus; (iii) entorhinal cortex, parahippocampal cortex and other temporal areas; (iv) striatum and thalamus and (v) middle frontal, cingular, parietal, insular cortices and pallidum. We concluded that this MRI scheme of atrophy progression in Alzheimer’s disease was close but did not entirely overlap with Braak staging of tauopathy, with a ‘reverse chronology’ between limbic and entorhinal stages. Alzheimer’s disease structural progression may be associated with local tau accumulation but may also be related to axonal degeneration in remote sites and other limbic-predominant associated proteinopathies.

## Teaser

Using extrapolated lifetime volumetric models of healthy and Alzheimer’s disease brain structures, Planche *et al*. propose a staging of atrophy progression in Alzheimer’s disease including (i) hippocampus and amygdala; (ii) middle temporal gyrus; (iii) entorhinal cortex and other temporal areas; (iv) striatum and thalamus and (v) middle frontal, cingular, parietal, insular cortices and pallidum.

## Graphical Abstract

**Graphical Abstract:**
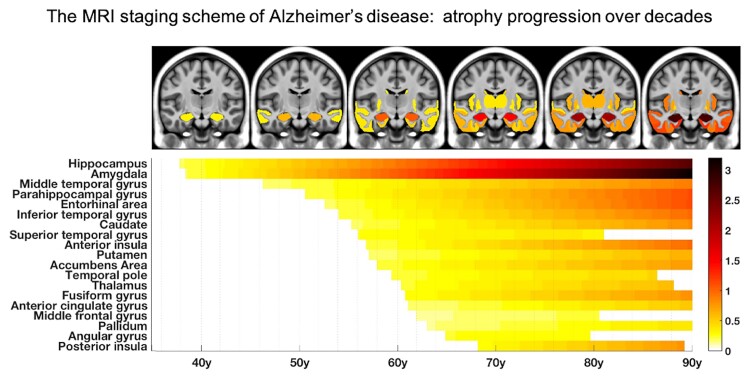

## Introduction

Alzheimer’s disease is a very slow progressing condition, likely to develop over three or four decades, from its preclinical phase to severe dementia and death.^1,2^ Unfortunately, to date, no longitudinal study has been long enough to describe such a slow progressing evolution through all these stages, and the model of Alzheimer’s disease progression over time remains partially hypothetical. However, providing insights into the spatiotemporal spreading of brain alterations, which will ultimately lead to the full spectrum of Alzheimer’s disease clinical symptoms, is crucial in the prospect of future therapeutic actions.

The current model of Alzheimer’s disease progression is mainly based on the Braak staging of Alzheimer’s disease tauopathy.^3^ This scheme was initially built on the concatenation of post-mortem pathological brain examinations from donors at all the stages of disease progression. Ongoing longitudinal tau-PET imaging studies support the Braak staging. However, the currently available follow-up is still relatively short, and only a few studies aimed at investigating the pre-symptomatic stages of the disease in cognitively unimpaired participants.^4^ Furthermore, first-generation tau-PET tracers do not allow the detailed investigation of medial temporal structures due to off-target binding in the choroid plexus, and PET-histological correlation studies showed that these tracers lack sensitivity and could only identify patients with Braak stages  ≥4.^5^ Although the accumulation of Alzheimer’s disease tauopathy usually precedes measurable atrophy,^6^ the anatomical progression of brain atrophy in Alzheimer’s disease may not perfectly match with tau topography and atrophy can experimentally precede overt cell loss.^7^

Indeed, many other factors are likely to explain brain atrophy in Alzheimer’s disease: the vascular pathology and the neuroinflammation coupled with Alzheimer’s disease neuropathological changes,^8^ interactions with other proteinopathies,^9^ network disruption due to local pathology combined with axonal degeneration and distant atrophy,^10^ local replication rate of tau aggregates^11^ and regional differential vulnerabilities to tau pathology and hypometabolism.^12^ Thus, the chronological progression over decades of neurodegeneration and brain atrophy in Alzheimer’s disease has never been formally depicted at the whole-brain level, and it is unknown whether it strictly follows the Braak staging of Alzheimer’s disease tauopathy.

Because no single longitudinal data over decades is available, we proposed to take advantage of BigData sharing in neuroimaging by analysing MRI databases of both Alzheimer’s disease patients and healthy subjects at different ages covering the entire lifespan. From these cross-sectional data, we processed a massive number of MRI to generate extrapolated lifespan models of several brain structure volumes evolution. Then, we validated such an approach by comparing profiles extrapolated from these cross-sectional data with independent available ‘truly’ longitudinal data. Finally, we described an MRI staging of volume loss in Alzheimer’s disease by describing the timing (and severity) of significant divergence between healthy subjects and Alzheimer’s disease patients’ volumetric trajectories.

## Methods

### Datasets

Normal and Alzheimer’s disease trajectories of brain atrophy were estimated thanks to the aggregation of nine open-access databases. The number of subjects included in the present study is provided after QC:

– *C-MIND:* 236 images of control subjects from the C-MIND dataset (https://research.cchmc.org/c-mind/) are used in this study. All the 3D T_1_-weighted (T_1_w) MPRAGE high-resolution MRI were acquired at the same site on a 3 T scanner with spatial resolution of 1 mm^3^ acquired using a 32 channel SENSE head-coil.
– *NDAR:* 382 of control subjects from the Database for Autism Research (NDAR) (https://ndar.nih.gov) are used in this study. The T_1_w 3D MRI were acquired on 1.5 T MRI and 3 T scanners. In our experiments, we used the NIHPD (http://www.bic.mni.mcgill.ca/nihpd/info/data_access.html) dataset and 197 images of control subjects from the Lab Study 19 of the National Database for Autism Research. For the NIHPD dataset, the 3D T_1_w spoiled gradient-recalled echo (SPGR) MRI were acquired at six different sites with 1.5 T systems by General Electric and Siemens Medical Systems with spatial resolution of 1 mm^3^. The 3D T_1_w MPRAGE MRI from the Lab Study 19 were scanned using a 3 T Siemens Tim Trio scanner at each site with spatial resolution of 1 mm^3^.
– *ABIDE:* 492 control subjects from the Autism Brain Imaging Data Exchange (ABIDE) dataset (http://fcon_1000.projects.nitrc.org/indi/abide/) are used in this study. The MRI are T_1_w MPRAGE acquired at 20 different sites on 3 T image and the details of the acquisition, informed consent and site-specific protocols are available on the website.
– *ICBM:* 294 normal subjects from the International Consortium for Brain Mapping (ICBM) dataset (http://www.loni.usc.edu/ICBM/) obtained through the LONI website are used in this study. The T_1_w MPRAGE MRI were acquired on a 1.5 T Philips GyroScan imaging system (Philips Medical Systems, Best, The Netherlands) with spatial resolution of 1 mm^3^.
– *IXI:* 549 normal control from the Information eXtraction from Images (IXI) database (http://brain-development.org/ixi-dataset/) are used in this study. The MRI are T_1_w images collected at three sites with 1.5 and 3 T scanners with spatial resolution close to 1 mm^3^.
– *ADNI1&2:* 404 control subjects and 332 Alzheimer’s disease patients from the Alzheimer’s Disease Neuroimaging Initiative (ADNI) database (http://adni.loni.usc.edu) Phases 1 and 2 are used in this study. These baseline MRI are T_1_w MPRAGE and SPGR acquired on 1.5 T scanners and 3 T at 60 different sites across the USA and Canada with reconstructed spatial resolution of 1 mm^3^. In ADNI 1&2, the diagnosis of Alzheimer’s disease was made according to NINCDS/ADRDA criteria for probable Alzheimer’s disease.^13^ Patients were included whatever their clinical presentation (typical or atypical Alzheimer’s disease), but all participants had abnormal memory functions at baseline, and Clinical Dementia Rating scale = 0.5 or 1. Alzheimer’s disease biomarkers analysis was optional. The complete list of inclusion/exclusion criteria is available here: http://adni.loni.usc.edu/methods/documents/.
– *AIBL:* 467 control subjects (857 images) and 83 Alzheimer’s disease patients (113 images) from the Australian Imaging, Biomarkers and Lifestyle (AIBL) database (http://www.aibl.csiro.au/) are used in this study. We used the longitudinal dataset as defined in Wen *et al*.^14^ and provided on the platform Clinica (http://www.clinica.run). These images are T_1_w acquired on 3 T MR scanners with the ADNI protocol (http://adni.loni.ucla.edu/research/protocols/mri-protocols) and with custom MPRAGE sequence on the 1.5 T scanners. In AIBL, the diagnosis of Alzheimer’s disease was made during clinical review panel meetings,^15^ according to NINCDS/ADRDA criteria for possible or probable Alzheimer’s disease .^13^

### Image processing

**Figure 1:**
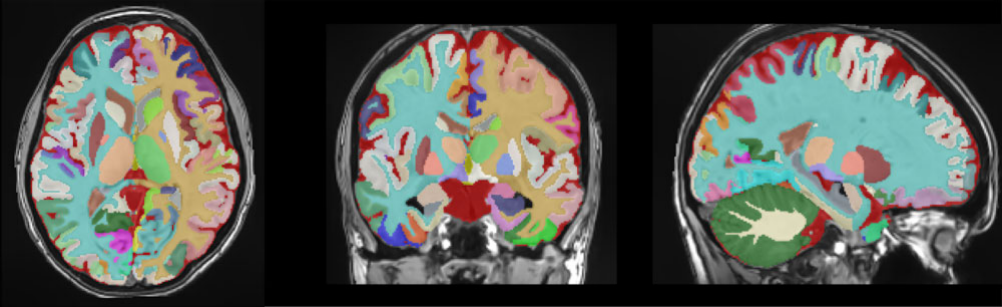
**Illustration of the AssemblyNet segmentation in the three axes**. It included the bilateral segmentation of 9 subcortical structures, 17 frontal gyri/lobules, 8 temporal gyri/lobules, 6 parietal gyri/lobules, 8 occipital gyri/lobules, 6 gyri in the limbic cortex and 5 sub-regions of the insular cortex, the ventricles and the cerebellum.

All the considered T_1_w MRI were processed with AssemblyNet.^16^ This software produces whole-brain segmentation of fine-grained structures using a large ensemble of deep neural networks. On the 132 structures produced by AssemblyNet following the Neuromorphometrics labels,^17^ we considered only 122 grey matter regions (61 left and 61 right). An illustration of the AssemblyNet segmentation is provided in Fig. 1. It included 9 subcortical structures, 17 frontal gyri/lobules, 8 temporal gyri/lobules, 6 parietal gyri/lobules, 8 occipital gyri/lobules, 6 gyri in the limbic cortex and 5 sub-regions of the insular cortex, ventricles and the cerebellum.

The AssemblyNet pipeline included the following steps. After denoising,^18^ images were corrected for inhomogeneity,^19^ affine-registered into the Montreal Neurological Institute (MNI) space using ANTS^20^ and tissue-based intensity normalized.^21^ Then, the intracranial cavity was segmented using the NICE method.^22^ Afterwards, structure segmentation was achieved using 250 U-Nets through a multi-scale framework.

A multi-stage quality control procedure was performed by P.C., blinded of the subject’s group, as previously described.^23,24^ First, a visual assessment was done for all input images by checking screen shots of one sagittal, coronal and axial slice in the middle of the 3D volume. Images were rejected if partial head coverage, motion artefact, high distortion or abnormal noise level was detected. Then, a visual assessment of processing quality was carried out using the segmentation report which provides screenshots for each step of the pipeline. Images were rejected after this step in case of inaccurate registration in the MNI space, inaccurate intracranial cavity extraction, missing brain structures or over/under-segmentation of brain structures. The last control was performed by individually checking of all outliers (values higher/lower than 2 SD of the estimated model). For each outlier, the segmentation map was inspected using a 3D viewer. In case of segmentation failure, the subject was removed from the study.

### Statistical analyses

To compensate for the variability introduced by head size difference, models were estimated on normalized volumes (% of total intracranial volume). Left and right volumes were added to obtain the final volume structure. Statistics were performed with MATLAB using default parameters.

Different strategies were considered to model the trajectories of each brain structure over time, as previously described.^24^ Briefly, the candidate models were tested from the simplest to the most complex: (i) a linear model, (ii) a quadratic model and (iii) a cubic model. A model was kept as a potential candidate only when simultaneously *F*-statistic based on ANOVA (i.e. model versus constant model) was significant (*P* < 0.05) and when all its coefficients were significant using *t*-statistic (*P* < 0.05). We finally used the Bayesian information criterion to compare the candidate models. This model selection procedure was applied to all the considered structures.

Afterwards, *z*-score and ‘distance’ between healthy and Alzheimer’s disease trajectories were computed on the estimated models. The prediction bounds were estimated with a confidence level of 95%. A brain structure was considered to be significantly smaller in Alzheimer’s disease compared with healthy ageing when the two structural trajectories diverged and when their 95% confidence intervals no longer overlapped.

To compare our lifespan cross-sectional models and longitudinal models over restricted periods, we followed a previously published strategy^25^ where cross-sectional and longitudinal models were compared using Spearman correlation over the age period defined by longitudinal models. We performed two experiments to analyse model similarities and atrophy pattern similarities. First, we estimated correlations between the models’ values for each structure. Second, we estimated the correlation between vectors containing cross-sectional volume shrinkage and longitudinal atrophy for all the structures simultaneously. These volume changes were estimated as the difference between the value at the starting age of longitudinal models and the final one.

Finally, the sequence of significant divergence of the different brain structures was listed in chronological order to obtain the MRI staging scheme.

### Data availability

MRI raw data from the different cohorts are available online (see Acknowledgments). Other data are available from the corresponding author upon reasonable request.

## Results

### Dataset description

To study structures’ trajectories of healthy controls and Alzheimer’s disease across the entire lifespan, we compiled several open-access databases to construct two datasets. Their composition and characteristics are described in Table 1. After QC, 3512 MRI from healthy controls and 415 from Alzheimer’s disease patients remained for the analyses. MRI from the longitudinal AIBL database (*n* = 113) were used as an external testing dataset to validate our models. Consequently, we used 2665 MRI from healthy controls to estimate our healthy lifespan models. To infer Alzheimer’s disease models over the entire lifespan, we combined 1874 healthy controls younger than 55 years of age (all the subjects younger than 55 in the 2665 that were used for healthy ageing models) and 332 Alzheimer’s disease patients older than 55 to build our lifespan Alzheimer’s disease models.

**Table 1:** Dataset description.

Dataset	*N*	Gender	Age (years)
**Healthy controls (total)**	**3512**	**−**	**−**
*C-MIND*	236	F = 129; M = 107	8.44 [0.74–18.86]
*NDAR*	382	F = 174; M = 208	12.39 [1.08–49.92]
*ABIDE*	492	F = 84; M = 408	17.53 [6.50–52.20]
*ICBM*	294	F = 142; M = 152	33.75 [18–80]
*IXI*	549	F = 307; M = 242	48.76 [20.0–86.2]
*OASIS*	298	F = 187; M = 111	45.34 [18–94]
*ADNI 1&2*	404	F = 203; M = 201	74.81 [56–90]
*Longitudinal AIBL (467 subjects)*	857	F = 485; M = 372	74.15 [60.5–92.4]
**Alzheimer’s disease (total)**	**415**	**−**	**−**
*ADNI 1&2*	332	F = 151; M = 181	75.13 [55–91]
*Longitudinal AIBL (83 subjects)*	113	F = 66; M = 47	73.35 [55.5–93.4]

This table provides the name of the dataset, the number (*n*) of considered images (after quality control), the gender proportion and the average ages and intervals in brackets.

### Identification of brain structures diverging between healthy subjects and Alzheimer’s disease patients trajectories

**Figure 2:**
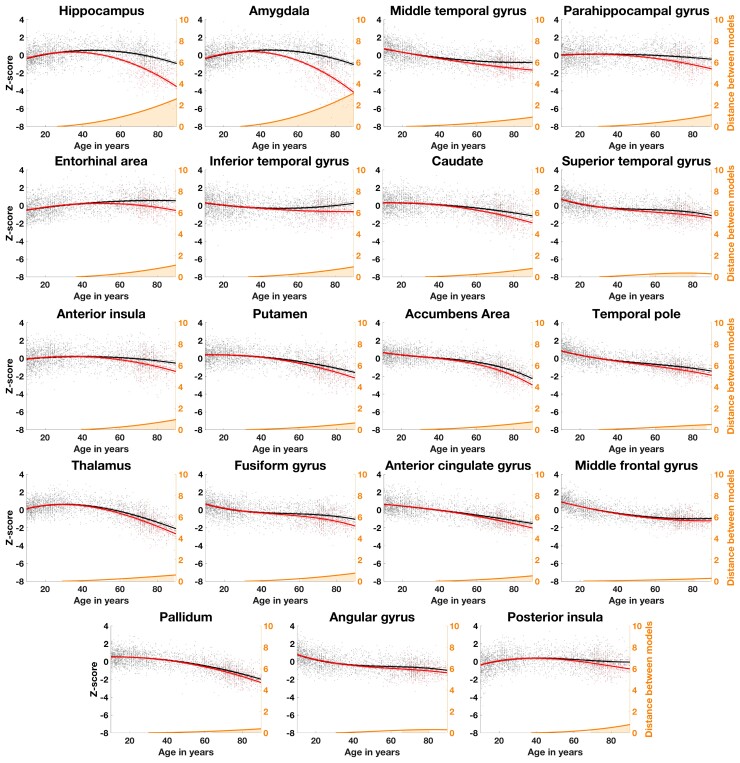
**Lifespan trajectories based on *z*-scores of normalized brain volumes for healthy ageing subjects (black line) and Alzheimer’s disease patients (red line)**. Black dots represent all healthy individuals and red dots Alzheimer’s disease patients. The prediction bounds of the models are estimated with a confidence level at 95%. The orange curve represents the distance between the healthy and pathological models. The orange area indicates the time period where the confidence intervals of both models do not overlap. Only models detected as significantly different between healthy ageing and Alzheimer’s disease are presented in this figure (19 brain structures out of 61 tested using AssemblyNet).

Figure 2 shows the 19 brain structures (out of the 61 grey matter structures we have tested using AssemblyNet) that significantly diverged during lifespan between Alzheimer’s disease and healthy ageing models. The theoretical starting age of statistically significant divergence between healthy and pathological models appears to be between 38 and 68 years old, depending on the structure. The most affected structures over time were the amygdala and the hippocampus (distance between healthy and ageing model at 90 years old = 3.20 and 2.65, respectively), followed by the entorhinal and parahippocampal cortices (distance = 1.18 and 1.13), the insula and the inferior temporal gyrus (distance = 0.96 and 0.96).

### Comparison of cross-sectional and longitudinal lifespan models

**Figure 3:**
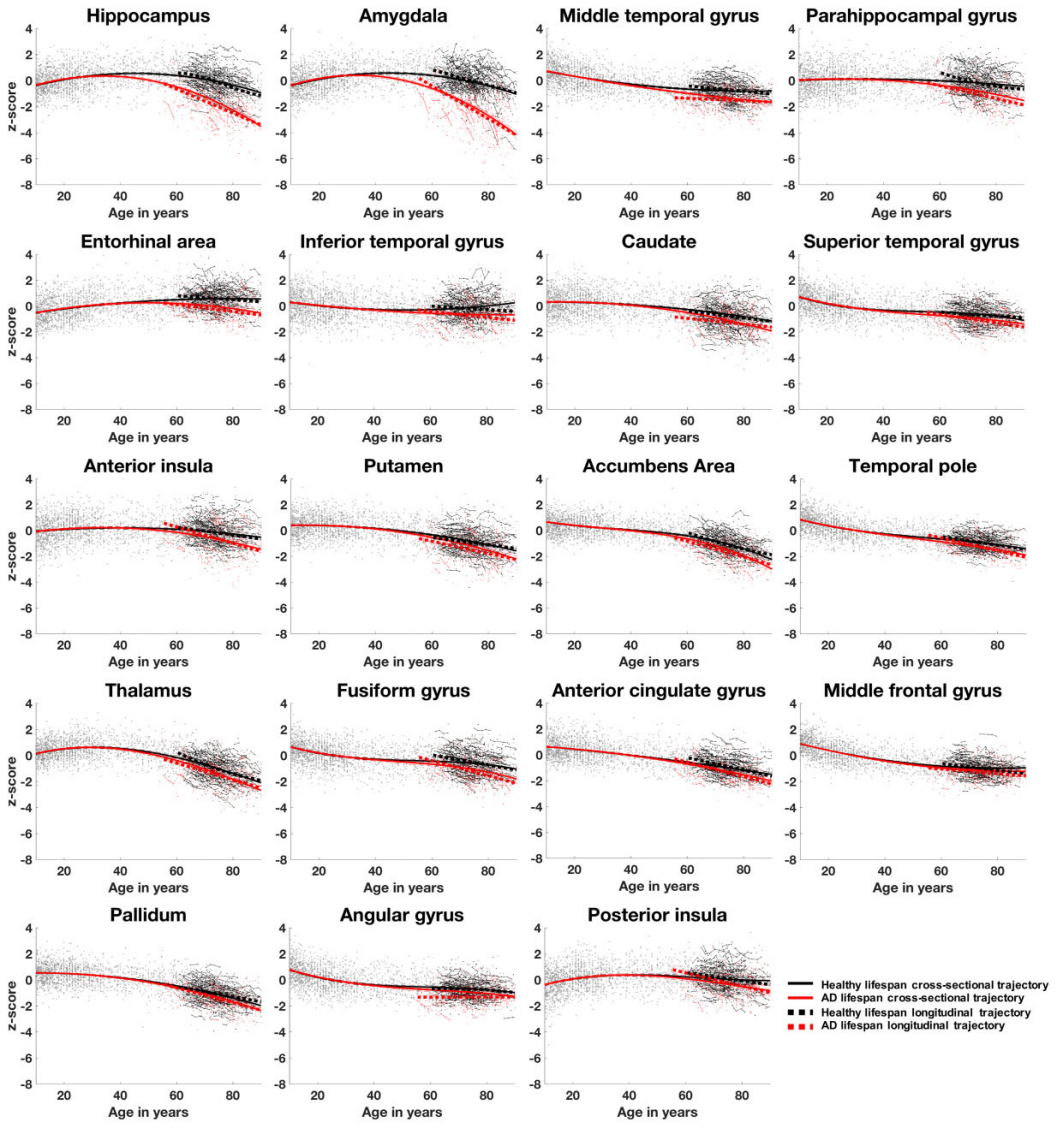
**Graphical comparison of cross-sectional (whole lines) and longitudinal (dotted lines) trajectories based on *z*-scores of normalized brain volumes for healthy ageing (black lines and dots) and Alzheimer’s disease patients (red lines and dots)**. Only the 19 structures detected as significantly different between healthy and pathological conditions are represented here. The average correlation coefficients for these 19 structures were 0.91 for Alzheimer’s disease models and 0.82 for healthy ageing models.

For all the 19 structures detected as significantly different between normal ageing and Alzheimer’s disease, we noticed a significant correlation between cross-sectional and longitudinal models (*P* < 0.05) in the age range of [55.5–93.4] for Alzheimer’s disease patients and [60.5–92.4] for healthy subjects. The average correlation between the models’ values over these 19 structures was 0.91 for Alzheimer’s disease models and 0.82 for healthy ageing models. Moreover, we found significant correlations between cross-sectional volume shrinkage and longitudinal atrophy for both Alzheimer’s disease and healthy ageing trajectories (*P* < 0.05). The atrophy pattern correlation for these 19 structures was 0.90 for Alzheimer’s disease models and 0.85 for healthy ageing models. Figure 3 shows the graphical congruence of models based on cross-sectional and longitudinal data on all the 19 affected structures.

### The MRI staging scheme

**Figure 4:**
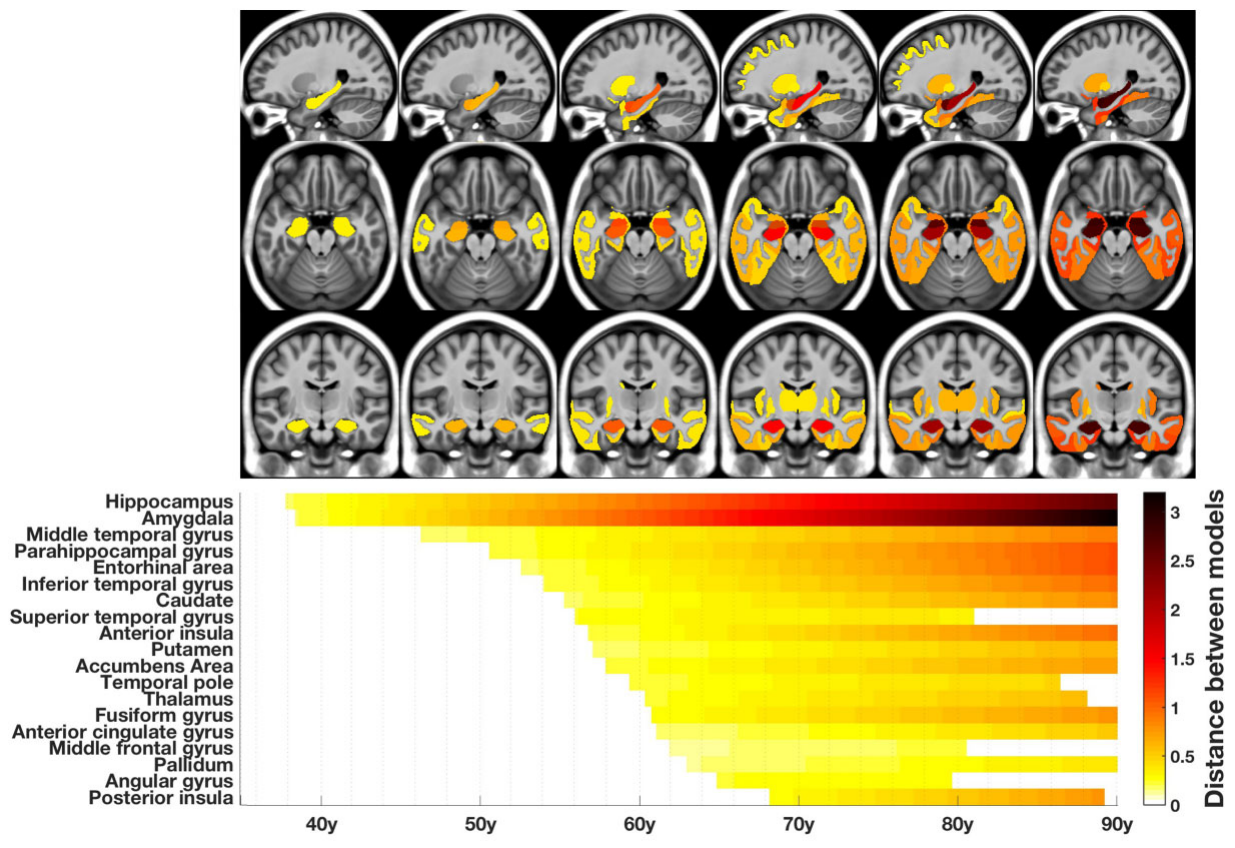
**The MRI staging scheme of structural progression of Alzheimer’s disease**. The figure represents the chronological progression of distances between healthy and Alzheimer’s disease trajectories. The severity of structural divergence is colour-coded according to the bar at the bottom right of the figure.

We then mapped the sequential divergence of healthy and Alzheimer’s disease trajectories in Fig. 4. Schematically, five major stages in Alzheimer’s disease structural progression appeared: (i) hippocampus and amygdala; (ii) middle temporal gyrus; (iii) entorhinal cortex, parahippocampal cortex and other temporal areas (inferior temporal gyrus, superior temporal gyrus, temporal pole and fusiform gyrus); (iv) thalamus and striatum (accumbens, caudate, putamen) and (v) middle frontal, anterior cingular, parietal (angular gyrus), insular cortices and pallidum.

## Discussion

In this study, we combined multiple large-scale MRI databases and whole-brain segmentation of fine-grained structures using a large ensemble of deep neural networks to describe the first exhaustive chronological structural progression of Alzheimer’s disease over decades and the differential severity of volumetric structure alterations. We finally proposed the following brain MRI staging scheme of structural progression in Alzheimer’s disease: (i) hippocampus and amygdala; (ii) middle temporal gyrus; (iii) entorhinal cortex, parahippocampal cortex and other temporal areas; (iv) striatum and thalamus and (v) middle frontal, cingular, parietal and insular cortices. The most severely affected structures during the entire course of Alzheimer’s disease were the amygdala and the hippocampus, followed by the entorhinal and parahippocampal cortices.

This MRI staging scheme of atrophy progression is close to but does not fully overlap with Braak staging of Alzheimer’s disease tauopathy.^3^ The main difference between these two staging models is the ‘reverse chronology’ between limbic and entorhinal stages (the earliest stages). Indeed, transentorhinal and entorhinal cortices are the first affected by tau neurofibrillary tangles (NFT) (Braak Stages I and II) before hippocampus [cornu ammonis 1 (CA1) pyramidal cells] and amygdala (Braak Stages III and IV). After these entorhinal and limbic stages, the sequential involvement of the thalamus and the striatum before the isocortical associative areas is concordant between MRI and Braak staging. However, Braak staging can be questioned. A recent 3D mapping of human tau pathology suggests, for instance, a more widespread distribution of NFT at the early stages of Alzheimer’s disease: similar levels of tau burden were found in entorhinal areas and the amygdala, the hippocampus, and the temporopolar cortex, supporting our MRI findings.^26^

Although tau distribution is usually correlated with focal atrophy in Alzheimer’s disease, recent tau-PET studies support our findings and the ‘early stage discrepancy hypothesis’ between atrophy and tau staging schemes. On the one hand, Mak *et al*.^27^ reported a disproportionate increase in tau accumulation in widespread regions compared to cortical atrophy that was restricted to the temporal areas during the same period. On the other hand, Harrison *et al.*^28^ reported that atrophy might exceed tau accumulation by including brain regions relatively unaffected by tauopathy. Furthermore, MRI-pathological studies failed to correlate the rate of hippocampal atrophy with early hippocampal tau NFT during ageing.^29^ In turn, the exceptional vulnerability of hippocampal CA1 pyramidal neurons to Alzheimer’s disease neuropathological changes^30^ may explain differential atrophy between the hippocampus and the entorhinal cortex over time.

Experimentally, when pathological tau is specifically expressed in the entorhinal cortex of transgenic mice, atrophy is described in the hippocampus, a remote unaffected brain region at the early stage of this model, through axon degeneration of the perforant pathway (major afference from the entorhinal cortex to the hippocampus).^7^ The pathological accumulation of tau oligomers is also known to impair axonal transport before NFT formation, inducing early axonal degeneration and the loss of synaptic transmission in the target structures.^31,32^ In the case of the amygdala, early atrophy may also be due to the presence of pathological tau in the perirhinal, postrhinal and transentorhinal cortices (Braak Stage I), which give rise to important afferent projections to the amygdala in primates.^33^ Finally, atrophy has to be considered as the result of complex cellular processes including cell death associated with tau accumulation, but also from axonal degeneration in remote sites. This diaschisis phenomenon may precede tau tangles in the limbic system, explaining the ‘early stage discrepancy’ between MRI and Braak staging.

In addition, regional atrophy in Alzheimer’s disease does not rely on a single biological process, but instead reflects the local impact of many different possible mechanisms leading to neurodegeneration. Mainly, limbic-predominant age-related TAR DNA-binding protein 43 encephalopathy (LATE) neuropathological changes are frequent during ageing and often associated with Alzheimer’s disease neuropathological changes. TAR DNA-binding protein 43 (TDP-43) inclusions are known to accelerate brain atrophy and mainly affect the amygdala (Stage 1) and the hippocampus (Stage 2). The frequent co-occurrence (up to 75% of cases) of these proteinopathies may explain the excess of limbic atrophy compared to entorhinal atrophy at the early stages of Alzheimer’s disease.^34^ Indeed, pathological-MRI correlation studies showed that the hippocampus and the amygdala were the most frequently affected regions in TDP-43 positive brains and that the magnitude of atrophy mediated by TDP-43 inclusions was similar to tau-related atrophy.^35^

From the conceptual point of view, it is essential to keep in mind that the present MRI staging scheme (such as the Braak staging scheme) only describes the most frequent ‘limbic-predominant’ and ‘typical’ progressions of Alzheimer’s disease.^36,37^ This model only describes the ‘average’ structural course of Alzheimer’s disease and does not apply to ‘atypical’ Alzheimer’s disease, such as logopenic primary progressive aphasia, posterior cortical atrophy or behavioural Alzheimer’s disease, which are driven by different patterns of atrophy and represent less common ‘extremes’ of the Alzheimer’s disease anatomical spectrum.^38^ Indeed, the methodology used in the present work does not take into account the recent description by Vogel *et al.*^39^ of distinct trajectories of tau deposition in Alzheimer’s disease. This work proposes an alternative to Braak’s model concerning tau spreading pattern, underlining the heterogeneity of Alzheimer’s disease development from one subject to another (not using pathological examination, but cross-sectional tau-PET with its limitations in terms of sensitivity). However, the most prevalent spatiotemporal trajectory of tau pathology was limbic in Vogel’s work, and spatial convergence was observed between Alzheimer’s disease trajectories. Furthermore, our MRI scheme of atrophy progression includes structures characterizing three of the four distinct trajectories of tau deposition, namely amygdala, hippocampus and entorhinal cortex (limbic trajectory), middle temporal gyrus, parahippocampal cortex and other temporal areas (lateral temporal trajectory) and parietal (angular gyrus) cortices (medial temporal lobe-sparing trajectory). Thus, our ‘average’ MRI staging scheme may not apply to all patients with Alzheimer’s disease, but to the greatest number, especially because cohorts such as ADNI 1&2 and AIBL included patients with ‘Alzheimer clinical syndrome’^40^ or ‘probable’ Alzheimer’s disease criteria^13,41^ (i.e. subjects with a single or multi-domain amnestic presentation, likely to have a limbic-predominant pattern of atrophy).

From the methodological point of view, we acknowledge that the estimated point of divergence between healthy and pathological trajectories remains hypothetical because of the lack of Alzheimer’s disease patients younger than 55 in the analysed cohorts. Another limitation of this study is that we cannot exclude that the anatomy of a structure of interest (the definition of boundaries on brain atlas, the precision of segmentation using MRI contrast, …) may impact the capability to depict volumetric modifications; this observation being particularly relevant for the entorhinal cortex.^42^ Thanks to an external validation using AIBL data, we have shown that our models based on cross-sectional ADNI data are reliable approximate of ‘true’ longitudinal trajectories, at least after 55 years old. Furthermore, the early atrophy in the amygdala and the hippocampus are consistent with a previous work from our group using a different method for structure segmentation.^24^ We also found substantial similarities with a previous article from another lab comparing the pattern of change of subcortical structures estimated on lifespan cross-sectional and longitudinal models in healthy ageing.^25^ Our study is, however, the first to assess the divergence between this healthy ageing trajectory and Alzheimer’s disease structural progression using inferred lifespan Alzheimer’s disease model, and at the whole-brain level.

To conclude, we have modelled the ‘average’ global structural progression of Alzheimer’s disease over the entire course of the disease. We found that the time course and the severity of structural alterations were close but did not completely overlap with Braak staging. We proposed a descriptive MRI staging scheme that will help better define the disease and interpret future studies looking at the differential vulnerabilities of brain structures to Alzheimer’s disease pathological processes.

## Abbreviations

ADNI =: Alzheimer’s Disease Neuroimaging Initiative
AIBL =: Australian Imaging, Biomarkers and Lifestyle
CA =: cornu ammonis
LATE =: limbic-predominant age-related TDP-43 encephalopathy
NFT =: neurofibrillary tangles
TDP-43 =: TAR DNA-binding protein 43
